# Loss of EMILIN-1/integrin axis drives microenvironmental reprogramming in gastric tumorigenesis

**DOI:** 10.1007/s10120-026-01781-4

**Published:** 2026-07-20

**Authors:** Alessandra Capuano, Maddalena Vescovo, Samanta Muzzin, Enrica Timis, Laura Cesaratto, Eliana Pivetta, Roberto Doliana, Antonio Palumbo, Renato Cannizzaro, Vincenzo Canzonieri, Eugenio Scanziani, Simone Canesi, Gustavo Baldassarre, Maurizio Mongiat, Paola Spessotto

**Affiliations:** 1 Molecular Oncology Unit, Centro Di Riferimento Oncologico Aviano, (CRO) IRCCS, Aviano, Italy; 2 Dipartimento Di Medicina, Università Degli Studi Di Udine, Udine, Italy; 3 Pathology Unit, Centro Di Riferimento Oncologico Aviano, (CRO) IRCCS, Aviano, Italy; 4 Oncological Gastroenterology Unit, Centro Di Riferimento Oncologico Aviano, (CRO) IRCCS, Aviano, Italy; 5 Department of Medical, Surgical and Health Sciences, University of Trieste, Trieste, Italy; 6 Dipartimento Di Medicina Veterinaria E Scienze Animali (DIVAS), Università Degli Studi Di Milano, Milan, Italy; 7 Mouse & Animal Pathology Lab (MAPLab), Fondazione UniMi, Milan, Italy

**Keywords:** Extracellular matrix, Gastric tumor microenvironment, Cancer associated fibroblasts, Gastric preneoplastic lesions, Paracrine communication

## Abstract

**Background:**

EMILIN-1 is an extracellular matrix glycoprotein with tumor-suppressive functions. While its loss is implicated in cancer progression, its specific role in the gastric tumor microenvironment and its clinical relevance remain poorly defined.

**Methods:**

Using in vitro cellular systems and genetically modified mouse models we investigated the consequences of impaired EMILIN-1 function on gastric epithelial transformation, stromal remodeling, and fibroblast reprogramming. Histopathological analysis, gene expression profiling, and functional assays were employed to assess phenotypic changes in epithelial, fibroblastic, and endothelial compartments.

**Results:**

We found that gastric cancer (GC) cells downregulate EMILIN-1 in stromal fibroblasts and lymphatic endothelial cells via paracrine signaling, leading to reduced EMILIN-1 deposition and disrupted lymphatic organization. Mechanistically, the interaction between EMILIN-1 and α4β1 integrin, which is absent in GC cells, modulates tumor cell proliferation; loss of this axis allows tumor cells to escape ECM-mediated growth control. Furthermore, EMILIN-1 downregulation reprograms fibroblasts into a pro-tumorigenic phenotype, which enhances GC cell migration and clonogenic potential. EMILIN-1 loss-of-function (E955A) mice showed increased susceptibility to pre-neoplastic lesions, a finding mirrored in human dysplastic tissues where EMILIN-1 was markedly reduced.

**Conclusion:**

Our findings establish EMILIN-1 as a master regulator of gastric tissue integrity. Its loss creates a tumor-permissive microenvironment by disrupting epithelial homeostasis, impairing lymphatic structure, and promoting fibroblast activation. The consistent reduction of EMILIN-1 in early human dysplasia highlights its potential as a novel stromal biomarker for early GC risk stratification. Moreover, restoring the EMILIN-1/integrin axis represents a promising therapeutic strategy to re-establish growth control and suppress tumor progression.

**Supplementary Information:**

The online version contains supplementary material available at https://doi.org/10.1007/s10120-026-01781-4.

## Introduction

Gastric cancer (GC) remains one of the leading causes of cancer-related mortality worldwide, with most patients diagnosed at advanced stages and facing poor overall prognosis despite recent therapeutic advances [1, 2]. Historically, GC research has primarily focused on epithelial transformation driven by *Helicobacter pylori* infection, chronic inflammation, and genomic instability [3]. However, accumulating evidence highlights the critical role of the tumor microenvironment (TME), comprising immune cells, blood and lymphatic vasculature, fibroblasts, and the extracellular matrix (ECM), in shaping tumor progression [4].

Beyond providing structural support, several ECM components are now recognized as active regulators of tumor behavior. These molecules influence key processes such as cell adhesion, growth factor signaling, and immune cell recruitment. Nevertheless, their specific contribution to gastric tumorigenesis remains poorly understood. One such molecule is EMILIN-1, a glycoprotein localized at elastin–collagen interfaces and abundantly expressed by stromal fibroblasts and lymphatic endothelial cells (LECs) [5, 6]. In other tissue contexts, EMILIN-1 has been shown to exert tumor-suppressive functions by limiting cell proliferation, modulating lymphangiogenesis, and regulating TGF-β signaling [7–11].

We have previously identified EMILIN-1 as a tumor-suppressive ECM component in the gastric mucosa whose loss results in lymphatic dysfunction and proliferative advantages that fuel tumorigenesis [12]. However, the mechanisms by which GC cells circumvent EMILIN-1-mediated restraint, and how its downregulation reshapes the surrounding stroma, remain unclear. Notably, GC cells exhibit epigenetic silencing of *ITGA4*, the gene encoding the α4 integrin subunit [13–15], which binds EMILIN-1 through its gC1q domain [16]. Promoter hypermethylation of *ITGA4* abolishes α4β1 integrin expression in tumor epithelial cells, thereby preventing their interaction with EMILIN-1 in the surrounding matrix.

In this study, we investigated whether EMILIN-1 exerts a broader regulatory role within gastric TME. Leveraging genetically engineered mouse models, human gastric specimens, and in vitro systems mimicking tumor-stroma interactions, we uncover a dual escape strategy adopted by GC cells: suppression of EMILIN-1 production in stromal fibroblasts and LECs and loss of its cognate receptor α4β1-integrin in epithelial cells. Collectively, these alterations enable GC to evade EMILIN-1 mediated stromal surveillance and foster the development of a tumor-permissive microenvironment.

## Materials and methods

### Chemicals and reagents

Human recombinant gC1q WT and E954A domains were prepared as previously described [16]. Primary antibodies for Western blot and immunofluorescence are listed in Supplementary Table 1. HRP-conjugated secondary antibodies (Bethyl) and Alexa Fluor conjugates (Invitrogen) were used for Western blot and immunofluorescence, respectively.

### Cell cultures

Cells were maintained with 10% FBS and 100 U/mL penicillin/streptomycin unless specified. GES-1 (courtesy of Prof. Marina de Bernard, University of Padua) and NCI-N87 (ATCC) were maintained in RPMI; Hs746T (ATCC) in DMEM + 1 mM sodium pyruvate; AGS (ATCC) in F-12 K; Human Dermal Lymphatic Endothelial Cells in EBM™-2 + EGM™-2 MV BulletKit™ (Lonza); Bj-5ta fibroblasts (ATCC) in 4:1 DMEM/M199 + 10% FBS + 0.01 mg/mL hygromycin B (Invitrogen); primary dermal fibroblasts (courtesy of Dr. Danilo Ranieri, St. Andrea Hospital, Rome, Italy) in DMEM + 10% FBS.

### Animals and in vivo procedures

C57BL/6 J WT mice (Charles River) and Emilin1-E955A knock-in (E955A-KI) (Polygene Transgenetics, Rumlang, CH), formerly referred as E933A-TG corresponding to the E954 in the human hortolog [9, 17–19], were maintained at CRO-IRCCS. All procedures complied with national regulations (authorization n°348/2023 PR). Tamoxifen (350 mg/kg body weight, 3 days) was intra-peritoneally injected or gavaged to induce gastric atrophy and Spasmolytic Polypeptide-Expressing Metaplasia (SPEM) [20]; stomachs were collected 3 days later. DMP-777 (350 mg/kg, DBA Italia) was gavaged daily for 3 days to ablate parietal cells [21]. Tissues were fixed in 10% formalin, transferred to 70% ethanol, paraffin-embedded, sectioned (5 µm), and stained with H&E. The immunohistochemistry process involved staining tissue samples for α-SMA using a standard protocol with antibody incubation, peroxidase detection, and hematoxylin counterstaining. Digital image analysis was then performed on the slides using QuPath software to quantify α-SMA-positive areas and ImageJ software to measure the average width of the *muscularis mucosae* for each sample.

### Immunostaining

Patient characteristics are detailed in Supplementary Table 2. For immunohistochemical (IHC) analysis of EMILIN-1, we selected representative paraffin-embedded sections from biopsy or mucosectomy specimens. After heat-induced antigen retrieval with 10 mM citrate buffer (pH 6.0), staining was performed using the As556 rabbit polyclonal antibody [22] and an UltraVision LP Detection System HRP DAB kit (Thermo Scientific, Waltham, USA). Slides were scanned at 10 × magnification, and EMILIN-1 expression was evaluated in the lamina propria of matched dysplastic and non-dysplastic mucosal areas. The analysis included 1 to 14 fields per dysplastic region. A semi-quantitative H-score (range 0–300) was assigned by multiplying the percentage of positive cells by the staining intensity (0 to 3 +). In the same areas, the expression of α-SMA was assessed using a semi-quantitative score ranging from 0 to 5 (0, absence of staining; 5 maximal staining).

### Cell transfection

AGS cells were transfected with FuGENE HD (Promega) using empty pcDNA or α4-pcDNA following previously described protocols [23]. Stable clones were selected and maintained in F-12 K + 10% FBS + 0.5 mg/ml G418.

### RT-PCR analysis

RNA was extracted with NucleoSpin RNA II (MachereyNagel), reverse-transcribed, and amplified with iQ SYBRGreen (Bio-Rad) using primers in Supplementary Table 3. qRT-PCR was performed with CFX96 and analyzed with CFX Maestro (Bio-Rad).

### CRISPR/Cas9 system

EMILIN-1 KO in Bj-5ta fibroblasts was generated using Cas9/sgRNAs spanning exon 1 (Supplementary Table 4) cloned into pLV hUbC-Cas9-T2A-GFP (AddGene) [24]. Transduced cells were sorted, sequenced (Illumina MiSeq), and validated with Integrative Genomics Viewer. Clones were screened by PCR, using specific primers (Supplementary Table 5).

### Western blot analysis

Cells were lysed using two methods: one with NP-40/PBS for extracellular matrix (ECM) proteins and another with RIPA buffer for total proteins. Protein concentrations were measured, and equal amounts were separated by SDS-PAGE. After transferring to membranes, proteins were detected using specific primary (listed in the Supplementary Table 1) and HRP-conjugated secondary antibodies, visualized by chemiluminescence, and quantified using imaging software.

### FACS analysis

Cells were incubated with PE-anti-integrins antibodies (α2, clone P1E6-C5, Biolegend; α4, clone 9F10, Biolegend; α5, clone REA686, Miltenyi Biotec; α6, clone GoH3, Biolegend; α9β1, clone Y9A2, Biolegend; αvβ3, clone 23C6, Miltenyi Biotec; β1, clone MAR4, BD Pharmingen; β4, clone 439-9B, BD Pharmingen) or with APC-anti α3 integrin (clone ASC-1, Biolegend) or isotype control and analyzed with LSRFortessa X-20 (BD Biosciences). Bj-5ta cells were sorted with BD FACSAria™ III and expanded.

#### Cell adhesion assay

The CAFCA (Centrifugal Assay for Fluorescence-based Cell Adhesion) assay was performed as previously described [25]. Briefly, PVC strips were coated with substrates (Collagen I/IV, Fibronectin, recombinant gC1q). Calcein AM-stained cells were seeded, synchronized by centrifugation, incubated, and analyzed using an Infinite M1000 fluorometer (TECAN). Adhesion was calculated as percentage of bound cells.

#### xCELLigence technology for adhesion and proliferation assays

Real-time monitoring of cell adhesion and proliferation was performed using the xCELLigence RTCA Single Plate system (Agilent) with E-plate VIEW 96 (Acea Bioscience). Experimental setup, data acquisition, and analysis were conducted using RTCA Software Pro. The Cell Index, based on electrical impedance, reflects cell number, attachment, and morphology over time. Plates were coated with ECM proteins or gC1q, seeded with cells, and impedance recorded (every 5 min for 20 h for adhesion; hourly for 5 days for proliferation).

#### Tube formation assay

Cultrex® BME (Trevigen) was polymerized in 96-well plates. Human dermal LECs (2 × 10^4^/well) were seeded with/without conditioned medium from gastric cells. Tube formation was imaged every 15 min for 8 h (Leica AF6000LX) and analyzed with Wimasis WimTube software (Onimagin Technologies SCA) to determine the number of branch sites/nodes, loops/meshes, and number or length of tubes formed.

#### Colony assay

Cells were seeded at low density (1,000 cells/well) in 6-well plates and allowed to adhere overnight in complete F-12 K medium (supplemented with 10% FBS and 1% Penicillin/Streptomycin) at 37 °C with 5% CO₂. The following day, the medium was replaced with conditioned medium (CM) from either wild-type (WT) or EMILIN-1 knockout (KO) BJ-5ta fibroblasts, supplemented with 10% FBS. After a 7-day incubation, the colonies were gently washed with PBS, fixed with 4% paraformaldehyde (PFA) for 10 min at room temperature, and stained with 0.05% crystal violet in 20% methanol for 30 min. Following a final rinse with distilled water and air-drying, colonies were imaged. The number and size of the colonies were quantified using ImageJ software (win64 version).

#### Wound healing

Cells were cultured to confluence in F-12 K medium supplemented with 10% FBS and 1% Penicillin/Streptomycin. A uniform scratch wound was generated in the monolayer using a 200 µL pipette tip. After two gentle washes with PBS to remove detached cells, the cells were incubated in a serum-free medium consisting of a 1:1 mixture of fresh F-12 K and conditioned medium (CM) from either WT or KO BJ-5ta fibroblasts. Wound closure was monitored using an inverted phase-contrast microscope (Nikon Eclipse TS100) at 4 × magnification. The wound area at each time point was quantified using ImageJ software (win64 version).

#### Statistical analysis

Graphpad Prism (version 7.02) was used to perform statistical analyses. One-way ANOVA or unpaired (or paired) *t*-test was applied to normally distributed data. *P* < 0.05 was considered significant. Data are shown as mean ± SD.

## Results

### The EMILIN-1/α4β1-integrin axis regulates gastric epithelial proliferation and is bypassed by cancer cells.

We previously established EMILIN-1 as a tumor-suppressive sentinel in the gastric microenvironment. Both EMILIN-1 knock-out (KO) and E955A-KI mice, the latter expressing a mutant EMILIN-1 unable to bind α4β1 integrin, exhibited increased tumor susceptibility when challenged with gastric tumor cells or the carcinogen N-methyl-N-nitrosourea (NMU) [12]. This implicated the EMILIN-1/α4β1 integrin interaction as a critical growth control mechanism.

**Fig. 1:**
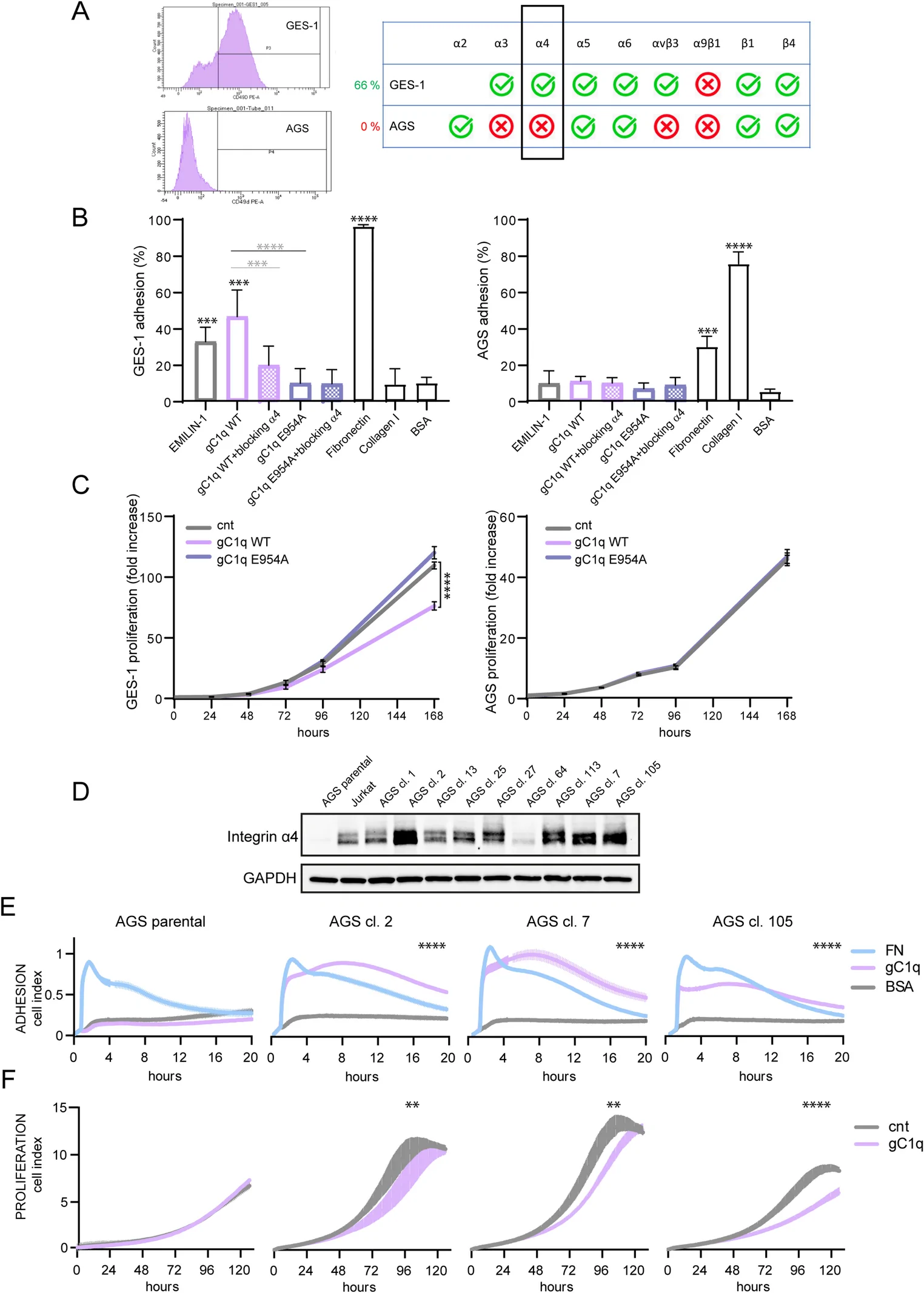
Role of α4 integrin in cell adhesion and proliferation of gastric cells. **A** Integrin expression profile in GES-1 and AGS cells, obtained from FACS analysis, showing the percentage of α4-positive cells. **B** Quantification of cell adhesion using CAFCA assay of GES-1 and AGS cells on various ECM substrates, expressed as the percentage of adherent cells. **C** Proliferation curves of GES-1 and AGS cells cultured in the presence of recombinant gC1q or its human mutant form E954A. **D** Western blotting of α4 expression in AGS stable clones. **E** Adhesion to gC1q and fibronectin, and **F** proliferation in the presence of gC1q of AGS parental cells and AGS clones (2, 7 and 105) stably expressing α4 integrin, detected by xCELLigence technology. **P < 0.01; ***P < 0.005; ***P < 0.001

Since α4-integrin is transcriptionally repressed in human GC epithelium but not in normal tissue [13], we hypothesized that cancer cells evade growth inhibition not only by depleting EMILIN-1 from their microenvironment [12] but also by losing the α4-integrin receptor itself. To assess this, we characterized the integrin expression profiles of normal GES-1 gastric mucosa cells and intestinal-type AGS GC cells. Flow cytometry revealed that while both lines expressed α5, α6, β1, and β4 subunits, only GES-1 cells expressed α3, α4, and αv integrins. Notably, α9-integrin, an EMILIN-1-binding homolog of α4 [7, 26], was absent in both (Fig. 1A, Fig. S1). This lack of α4 and α9 was a consistent feature across other GC lines (Fig. S2), confirming that disruption of the EMILIN-1/integrin axis is a common event in gastric carcinogenesis.

Integrin activation was assessed through adhesion assays using various ECM substrates. In line with the 66% expression of α4-integrin, approximately 50% of GES-1 cells adhered specifically to the gC1q domain of EMILIN-1. This adhesion was abrogated by function-inhibiting antibodies targeting α4-integrin, and the human gC1q E954A mutant, which is unable to bind integrins [27], did not support adhesion (Fig. 1B).

Cell proliferation assays showed that the growth of GES-1 cells, which expresses α4β1-integrin, was modulated by the presence of gC1q. In contrast, AGS cells, lacking α4-integrin, did not exhibit any change in proliferation (Fig. 1C). Similarly, gC1q failed to reduce the proliferation of other GC cell lines deficient in α4-integrin (Fig. S2D).

To further explore this mechanism, AGS cells were transfected with α4-integrin, and the resulting clones were evaluated in adhesion and proliferation assays (Fig. 1D). Remarkably, α4-expressing AGS cells exhibited enhanced and sustained binding to gC1q exceeding their adhesion to fibronectin used here as a control ECM protein (Fig. 1E). Furthermore, proliferation of these transfected clones was significantly modulated by gC1q, confirming that the α4β1-integrin/EMILIN-1 interaction directly influences the growth of GC cells (Fig. 1F). These findings demonstrate that α4β1-integrin is essential for EMILIN-1–mediated epithelial restraint and that its loss enables GC cells to bypass ECM-dependent proliferation control.

### Gastric Cancer cell–derived paracrine signals suppress EMILIN-1 in stromal compartments

We previously found that the deposition of EMILIN-1 was significantly decreased in malignant samples and that the presence of neutrophil elastase, a protease known to process EMILIN-1, could be responsible for the reduced levels of the protein [12]. To test whether the reduced EMILIN-1 protein levels were not only the result of a degradation process but also a consequence of transcriptional regulation, we developed an in vitro model designed to mimic the TME. The primary aim was to determine whether GC epithelial cells influence EMILIN-1 expression in stromal fibroblasts and LECs, which are known to be its main producers (Fig. S3A).

**Fig. 2:**
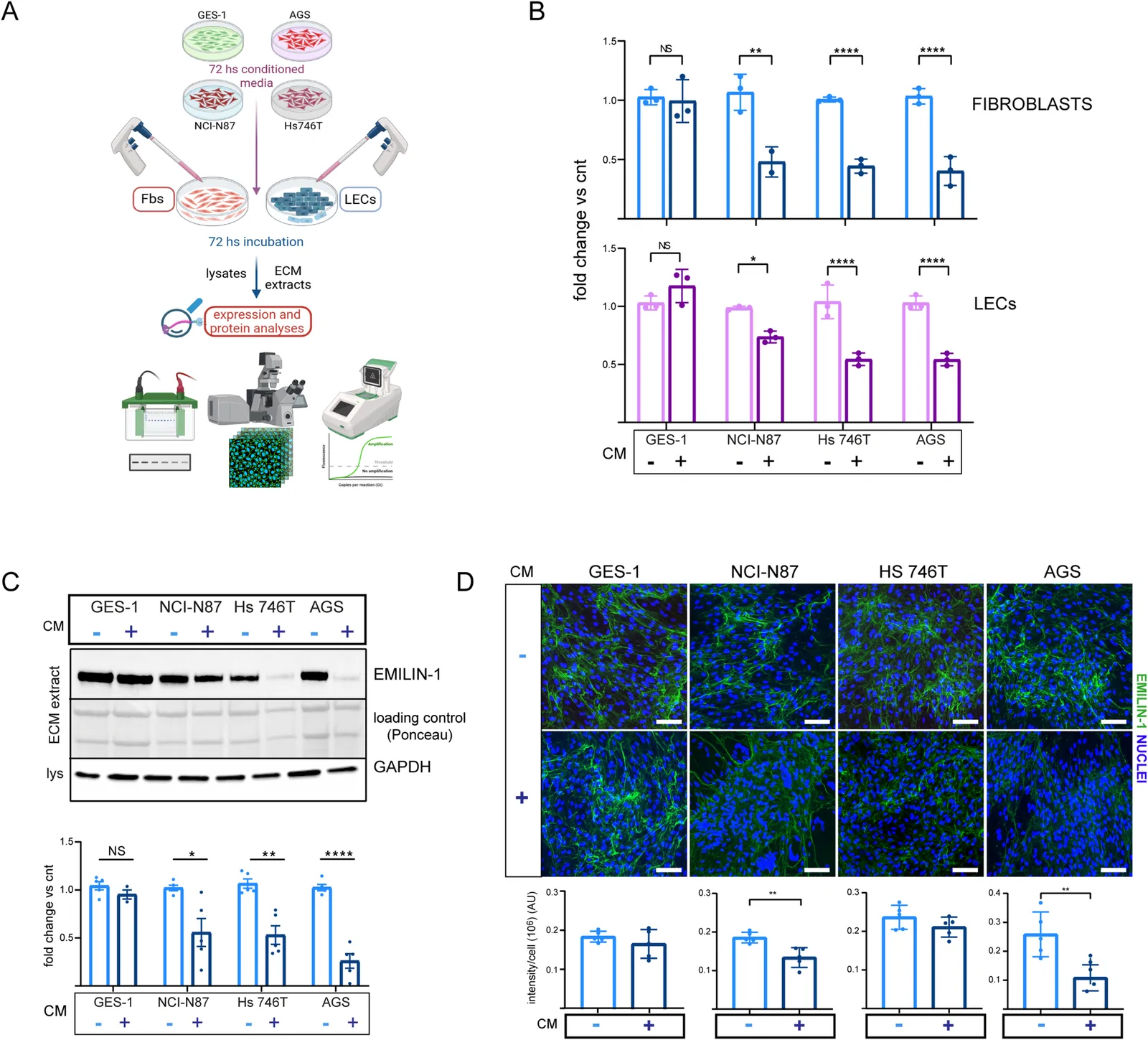
EMILIN-1 is downregulated by GC cells. **A** Schematic overview of experimental design. Stromal cells, fibroblasts (Fbs) and lymphatic endothelial cells (LECs), were cultured for 72 h with conditioned media (CM) derived from gastric epithelial cell lines. Control conditions included standard growth medium for each cell type. After treatment, ECM-enriched extracts, slides, and total RNA were collected for analysis of EMILIN-1 expression. The gastric cell lines used included GES-1 (non-tumorigenic), NCI-N87 (metastatic), Hs746T (diffuse-type GC), and AGS (intestinal-type GC). **B** RT-PCR analysis showing EMILIN1 mRNA downregulation in fibroblasts (top) and LECs (bottom) following CM treatment. **C** Western blot analysis of EMILIN-1 protein levels in ECM extracts from fibroblasts exposed to CM from GC cell lines. **D** Immunofluorescence analysis of fibroblasts treated with cancer-derived CM reveals decreased EMILIN-1 deposition (green). Quantification of EMILIN-1–positive signal was performed over the entire 20 × fields. At least five fields were analyzed per sample. Statistical analysis was performed using a two-tailed Student’s *t*-test. *P < 0.05, **P < 0.01, ****P < 0.001. Scale bar: 100 μm

To this end, stromal fibroblasts were exposed to CM collected from various GC cell lines: NCI-N87 (a metastatic line), Hs746T (representative of the diffuse histotype), and AGS (intestinal histotype). CM from normal gastric epithelial cells (GES-1) served as controls. Following treatment, EMILIN-1 levels in fibroblasts were assessed by RT-PCR and IF (Fig. 2A). EMILIN-1 mRNA expression was downregulated both in fibroblasts and LECs when cultured in the presence of CM derived from GC cells while CM from normal gastric epithelial cells had no effect (Fig. 2B). As shown in Fig. 2C, this downregulation led to a marked reduction of EMILIN-1 protein levels in ECM extracts. Immunofluorescence staining of fibroblast cultures incubated with GC-derived CM, particularly from AGS cells, confirmed a pronounced decrease in EMILIN-1 deposition (Fig. 2D).

To evaluate the specificity of EMILIN-1 downregulation, we also examined the expression of fibronectin, another major ECM component. Interestingly, both fibronectin mRNA and protein levels were not decreased by CM from GC cell lines; on the contrary they were significantly elevated (Fig. S3B, C).

To determine whether this regulatory mechanism was specific to GC or indicative of a broader cancer-associated mechanism, we exposed fibroblasts to CM from colon and ovarian cancer cell lines. Unexpectedly, we observed no reduction in EMILIN-1 mRNA levels; on the contrary, an opposite trend was detected (Fig. S3D).

Collectively, these findings indicate that EMILIN-1 downregulation induced by GC cells is predominantly driven through mechanisms affecting transcription and/or mRNA stability. While the contribution of degradative enzymes, such as matrix metalloproteinases (MMPs), cannot be completely excluded, our data argues against a major role for proteolytic loss in this context. Specifically, MMP expression in fibroblasts remained unchanged following treatment with tumor-CM, and no increase in MMP levels was observed in GC-derived CM compared to controls (data not shown). This strongly suggests that transcriptional suppression and/or mRNA destabilization, rather than enhanced ECM degradation, represent the predominant mechanism adopted by cancer cells to deplete EMILIN-1 in the gastric TME.

### EMILIN-1 downregulation promotes tumor progression through lymphatic dysfunction and stromal fibroblast activation

**Fig. 3:**
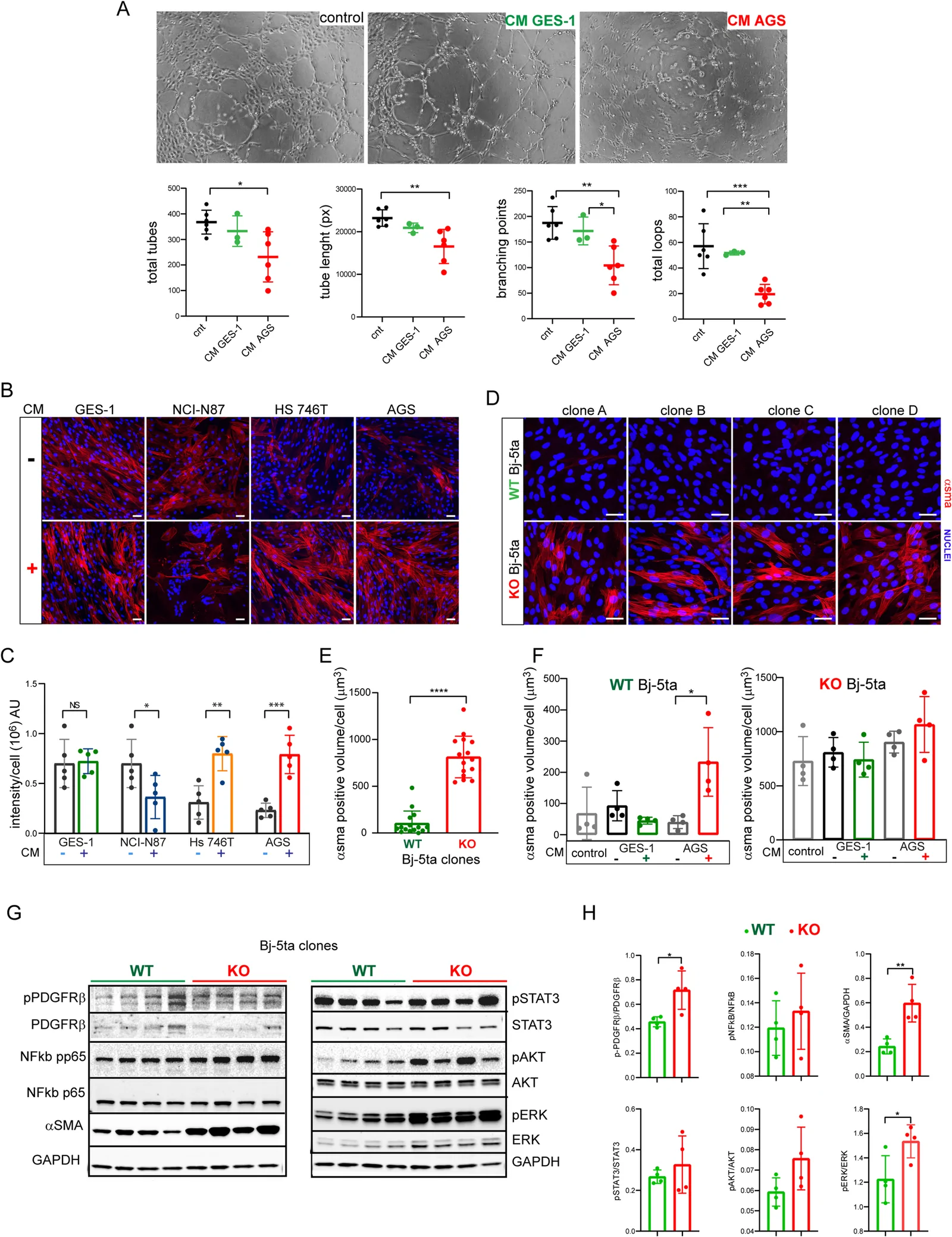
Conditioned media from GC cells induce a shift towards a Cancer-Associated Fibroblast (CAF)-like phenotype. **A** CM from AGS cells, but not from normal GES-1 cells, significantly impaired LEC tube formation. Brightfield images were analyzed with the Wimasis™ tool; quantification includes total tubule length, number of branching points, loops, and tubes. **B** Immunofluorescence analysis shows a marked increase in α-smooth muscle actin (α-SMA, red) expression in fibroblasts treated with conditioned media (CM) from GC cell lines. **C** Quantification of α-SMA–positive signal was performed across entire 20 × fields; at least five fields were analyzed per sample. **D** Representative images of genetically engineered KO fibroblasts clones exhibiting a myofibroblastic phenotype, with increased α-SMA deposition. **E** Quantification of α-SMA positive volume in the entire section field examined. For each clone (more than ten clones for each genotype), at least five fields were analyzed. Dots represent the mean value of different clones. **F** Comparison of α-SMA–positive volume in WT and KO Bj-5ta fibroblasts treated with CM from either GES-1 or AGS cells. Quantification was based on analysis of at least five fields per clone; each dot represents the mean value per clone (n = 4). **G** Representative western blots of lysates from Bj-5ta fibroblasts probed for the indicated proteins, including pPDGFRβ, pSTAT3, NF-κB p65, ERK, AKT, and α-SMA **H** Quantification of band intensity. Signals for phosphorylated proteins were normalized to their respective total protein and α-SMA to GAPDH used as loading control. Data are presented as mean ± SD. Statistical significance was assessed using two-tailed Student’s t-test (*P < 0.05, **P < 0.01, ***P < 0.001, ****P < 0.0001). Scale bar: 25 μm

CM from GC cell lines altered LEC behavior, as demonstrated by impaired tube formation in vitro. In contrast, CM from normal gastric cells (GES-1) did not affect LEC tube formation (Fig. 3A). Quantitative analysis revealed a significant reduction in total tube length, number of branching points, loops, and overall tube structures (Fig. 3A). These data identify a novel link between EMILIN-1 downregulation and LEC dysfunction in GC. This connection is part of a broader mechanism in which tumor-derived factors suppress EMILIN-1 and drive extensive remodeling of the ECM and suggests that by reshaping the stromal microenvironment in this way, including the active contribution of altered lymphatic vessels [12, 28], GC enhances its own aggressiveness and progression.

To investigate the functional impact of EMILIN-1 downregulation in fibroblasts, we examined whether EMILIN-1 loss promotes stromal activation toward a CAF-like/myofibroblastic phenotype. We first assessed the expression of α-smooth muscle actin (α-SMA), a marker commonly associated with activated myofibroblastic CAF subsets. EMILIN-1 downregulation significantly increased α-SMA levels (Fig. 3B,C), indicating a myofibroblastic transdifferentiation that is known to support tumor progression [29].

To test the cell-autonomous role of EMILIN-1 in promoting a CAF-like state, we generated a stable EMILIN-1 knockout (KO) in Bj-5ta fibroblasts using CRISPR-Cas9 (Fig. S4). Consistent with our initial findings, EMILIN-1 KO fibroblasts exhibited elevated basal α-SMA levels compared to WT controls (Fig. 3D,E). When stimulated with AGS-CM, WT fibroblasts robustly upregulated α-SMA, whereas KO fibroblasts, already primed toward an activated state, showed no further induction (Fig. 3F). Western blot analysis not only confirmed the significant increase in α-SMA protein but also revealed concomitant activation of key pro-survival and inflammatory signaling pathways, including AKT, ERK, NF-κB, and STAT3, in KO cells (Fig. 3G,H). Together with the observed upregulation of *CTGF* in KO clones (Fig. S4), these molecular changes align with established signaling networks involved in GC-associated fibroblast activation [30, 31].

**Fig. 4:**
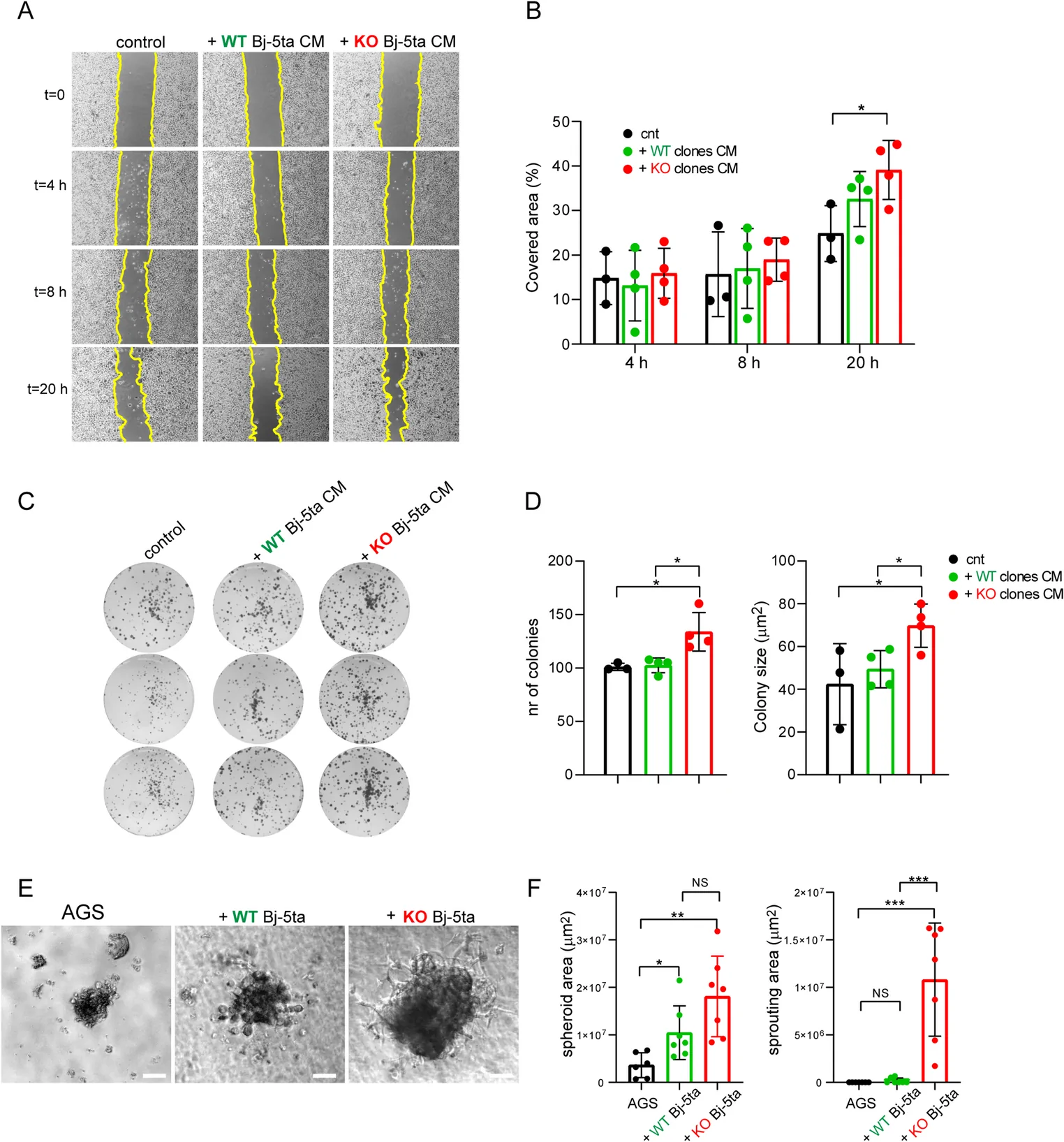
EMILIN-1-deficient fibroblasts promote AGS cell migration, invasive sprouting and clonogenicity. **A**, **B** Conditioned medium (CM) from KO fibroblasts accelerates AGS cell wound closure. **A** Representative images of the scratch assay at 0, 4, 8, and 20 h. **B** Quantification of wound closure over time. CM from four independent fibroblast clones per genotype was tested **C**, **D** CM from KO fibroblasts increases the clonogenic potential of AGS cells. **C** Representative images of colonies. **D** Quantification of colony number and size. Data are presented as mean ± SD. **E** 3D spheroids composed of AGS cells alone or co-cultured with WT or KO BJ-5ta fibroblasts were embedded in Matrigel and cultured for 5 days. Representative images are shown. **F** Graphs report quantification of spheroid size and sprouting-associated invasive area. Statistical significance was determined by a two-tailed Student's t-test (*P < 0.05, **P < 0.01, ***P < 0.001). Scale bar: 50 µm.

This EMILIN-1-driven phenotypic shift had profound functional consequences for GC cells. CM from EMILIN-1 KO fibroblasts significantly enhanced the migration of AGS and Hs746T cells in both scratch wound and haptotaxis assays (Fig. 4A,B; Fig. S5; Fig. S6A-C) and boosted AGS clonogenic potential (Fig. 4C,D). Moreover, 3D spheroid assays showed that co-culture with EMILIN-1 KO fibroblasts increased spheroid size and sprouting-associated invasion of both AGS (Fig. 4E,F) and Hs746T GC cells compared with WT fibroblasts (Fig. S6D,E), further supporting a pro-invasive stromal phenotype induced by EMILIN-1 loss.

In summary, our results demonstrate that loss of EMILIN-1 is sufficient to promote fibroblast activation toward a CAF-like/myofibroblastic state, characterized by pro-tumorigenic signaling and functional tumor-supportive properties. This reprogramming functionally enhances GC cell aggressiveness, revealing a critical role for EMILIN-1 in maintaining fibroblast homeostasis and restraining tumor-promoting microenvironmental remodeling.

### Impaired EMILIN-1 function enhances susceptibility to gastric preneoplastic transformation

**Fig. 5:**
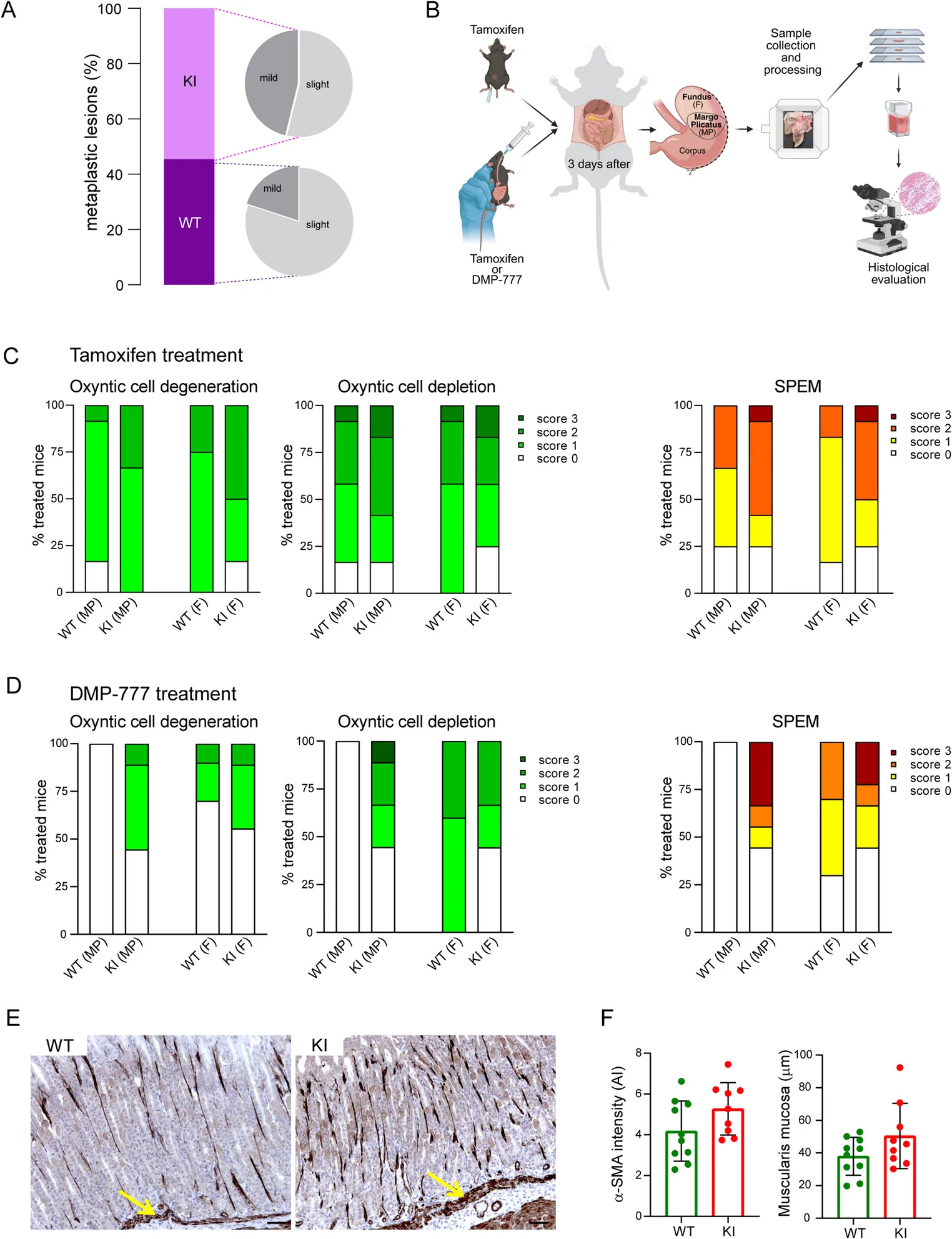
EMILIN-1 loss of function promotes increased incidence of gastric preneoplastic lesions. **A** Percentage of adult mice exhibiting spontaneous gastric metaplasia. For each genotype, the distribution of mild and moderate metaplastic alterations is indicated. **B** Experimental timeline and schematic overview of Tamoxifen or DMP-777 treatment protocols, including sample collection and histological processing. **C**-**D** Quantitative analysis of oxyntic cell loss severity scores and SPEM severity across genotypes in fundic mucosa **F** and in fundic mucosa at the border with the forestomach (*margo plicatus*) (MP) following oral gavage treatment with Tamoxifen (C, n = 11 WT and 12 KI) or DMP-777 (D, n = 6 WT and 16 KI). **E** Representative micrographs of α-SMA immunostaining in gastric mucosal sections from DMP-777-treated WT and KI-955A mice. Yellow arrows indicate the muscularis mucosae. The associated graphs **F** provide quantification of α-SMA immunoreactivity and muscularis mucosae thickness. Scale bar, 50 µm

To further explore whether genetically induced EMILIN-1 dysfunction contributes to the establishment of a tumor-permissive microenvironment, we analyzed the gastric mucosa of adult mutant mice. As shown in Fig. 5A, E955A-KI mice developed a modestly increased number of spontaneous metaplastic lesions compared to WT controls, but with a significantly higher proportion exhibiting more severe histological alteration scores. These findings suggested that the regenerative response is exacerbated in the absence of functional EMILIN-1–integrin signaling, underscoring the role of EMILIN-1 in maintaining mucosal homeostasis and restraining pathological remodeling. Importantly, these effects are independent of TGF-β activity, as its regulation remained unaltered in the mutant model [9, 17]. To further challenge epithelial integrity, we used Tamoxifen, which induces parietal cell loss, or DMP-777, a selective neutrophil elastase inhibitor known to trigger gastric pre-neoplastic lesions [32] and used here as a chemical approach to preserve EMILIN-1 integrity by preventing its proteolytic inactivation [33, 34] (Fig. 5B). Histological analyses were performed to assess both the incidence and severity of gastric lesions. Intraperitoneal injection of Tamoxifen caused a marked systemic toxicity, characterized by severe pathological alterations in the kidney and colon across all genotypes, without notable differences in the extent of parietal (oxyntic) cell loss in the gastric mucosa. Spasmolytic polypeptide-expressing metaplasia (SPEM) was not detected in any group, although higher scores for the presence of nuclear debris within glandular lumina and signs of regenerative hyperplasia were observed in E955A-KI mutant mice (Fig. S7). To reduce off-target toxicity, we switched to oral gavage administration. Under these conditions, Tamoxifen robustly induced oxyntic cell depletion and SPEM across all genotypes (Fig. 5C). While the differences were not statistically significant, the severity of metaplastic lesions appeared higher in E955A-KI mice compared to WT controls, suggesting increased susceptibility in the context of impaired EMILIN-1–integrin interaction (Fig. 5C). Notably, Tamoxifen-induced inflammation leads to neutrophil elastase release [35], which can degrade EMILIN-1 even in WT mice, partially reproducing the EMILIN-1-deficient environment of KI animals and thereby exacerbating lesion severity. Remarkably, treatment with DMP-777 resulted in more extensive pre-neoplastic alterations in EMILIN-1 mutant KI mice compared with their WT counterparts. While very few WT animals developed only focal and relatively mild lesions, a great percentage of KI treated mice displayed widespread mucosal damage accompanied by an increased number of metaplastic glands and higher histological alteration scores (Fig. 5D). These lesions were characterized by enhanced epithelial remodeling and expansion of atypical glandular structures, features indicative of a more sustained and dysregulated regenerative response. The differential susceptibility to DMP-777–induced injury strongly suggests that functional EMILIN-1 is required to limit the severity of epithelial transformation and to preserve tissue architecture under conditions of parietal cell loss. Together, these findings support the concept that EMILIN-1 acts as a stromal safeguard, buffering the gastric mucosa against pathological remodeling and reducing the likelihood of progression toward pre-neoplastic states.

**Fig. 6:**
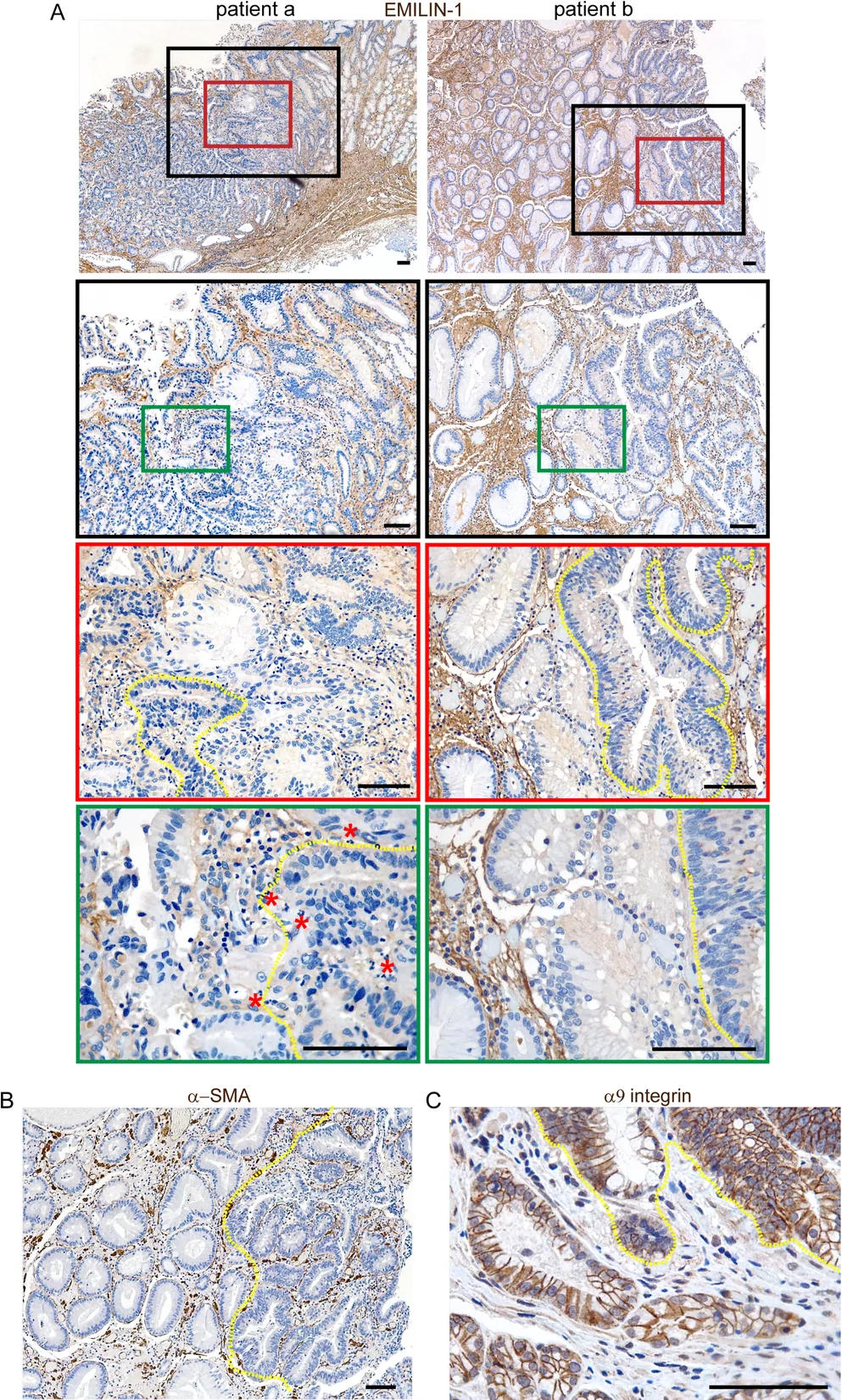
Loss of EMILIN-1 in gastric dysplasia. **A** Representative immunohistochemical staining for EMILIN-1 in human gastric dysplasia. Higher-magnification views (green, red, and black rectangles) show the loss of the intense fibrillar staining present in the normal mucosa within dysplastic foci. **B** Representative α-SMA immunostaining (patient b) showing similar staining intensity in normal and dysplastic areas. **C** Representative α9 integrin staining showing the strong membrane signal both in normal and dysplastic epithelium. The dashed yellow lines demarcate the normal and dysplastic areas. The red asterisks in (A) indicate the presence of neutrophils in dysplastic areas. Scale bar, 100 µm

Although the increase in α-SMA expression and muscularis mucosae width in E955A-KI mice was not statistically significant, the clear upward trend for both measures (Fig. 5E,F) directionally supports the in vitro finding that EMILIN-1 deficiency reprograms fibroblasts into α-SMA-positive CAF-like/myofibroblastic state. In human gastric dysplasia, EMILIN-1 was present in normal mucosa but absent in dysplastic regions (Fig. 6A and Fig. S8A), mirroring the mutant mouse phenotype. This indicates stromal remodeling as an early, active contributor to progression. The lack of a concurrent α-SMA increase in these human samples (Fig. 6B and Fig. S8B) further suggests that EMILIN-1 loss is a very early event, initiating stromal changes prior to full fibroblast reprogramming. Although α4 integrin could not be reliably assessed because of inadequate antibody performance, α9 integrin staining showed no evident differences between normal and dysplastic regions (Fig. 6C and Fig. S8C). These findings further point to EMILIN-1 downregulation as an early event in gastric dysplastic transformation. We also interrogated datasets for ITGA4 and ITGA9 expression in bulk GC samples. Because α4 integrin is expressed not only by epithelial cells but also by several inflammatory and immune cell populations infiltrating the TME, transcriptomic analyses of whole tumor tissues may partially obscure tumor-cell-specific downregulation, potentially explaining why no significant differences in α4 integrin expression were observed between normal and tumor tissues in our analyses (Fig. S9A). By contrast, α9 integrin expression was significantly reduced in tumor samples (Fig. S9A). This finding was further supported by analysis of the online TNMplot dataset, which showed a significant overall decrease in ITGA9 expression across several tumor types, including GC (Fig. S9B,C). Importantly, high EMILIN1 expression was associated with improved clinical outcome in GC patients (Fig. S8D,E). Kaplan–Meier analyses showed significantly longer OS and FP in patients with high EMILIN1 expression compared with those with low expression. Median OS was 26.2 versus 18.6 months (*P* = 0.031), whereas median FP was 17.3 versus 9.8 months (*P* = 0.0039). Stratification by tumor stage did not reveal statistically significant differences, although a trend toward improved survival was observed in patients with early-stage disease (Fig. S9E). Overall, these findings support a role for early EMILIN-1 loss in gastric tumorigenesis and link disruption of the EMILIN-1/integrin axis to disease progression and unfavorable clinical outcome.

## Discussion

**Fig. 7:**
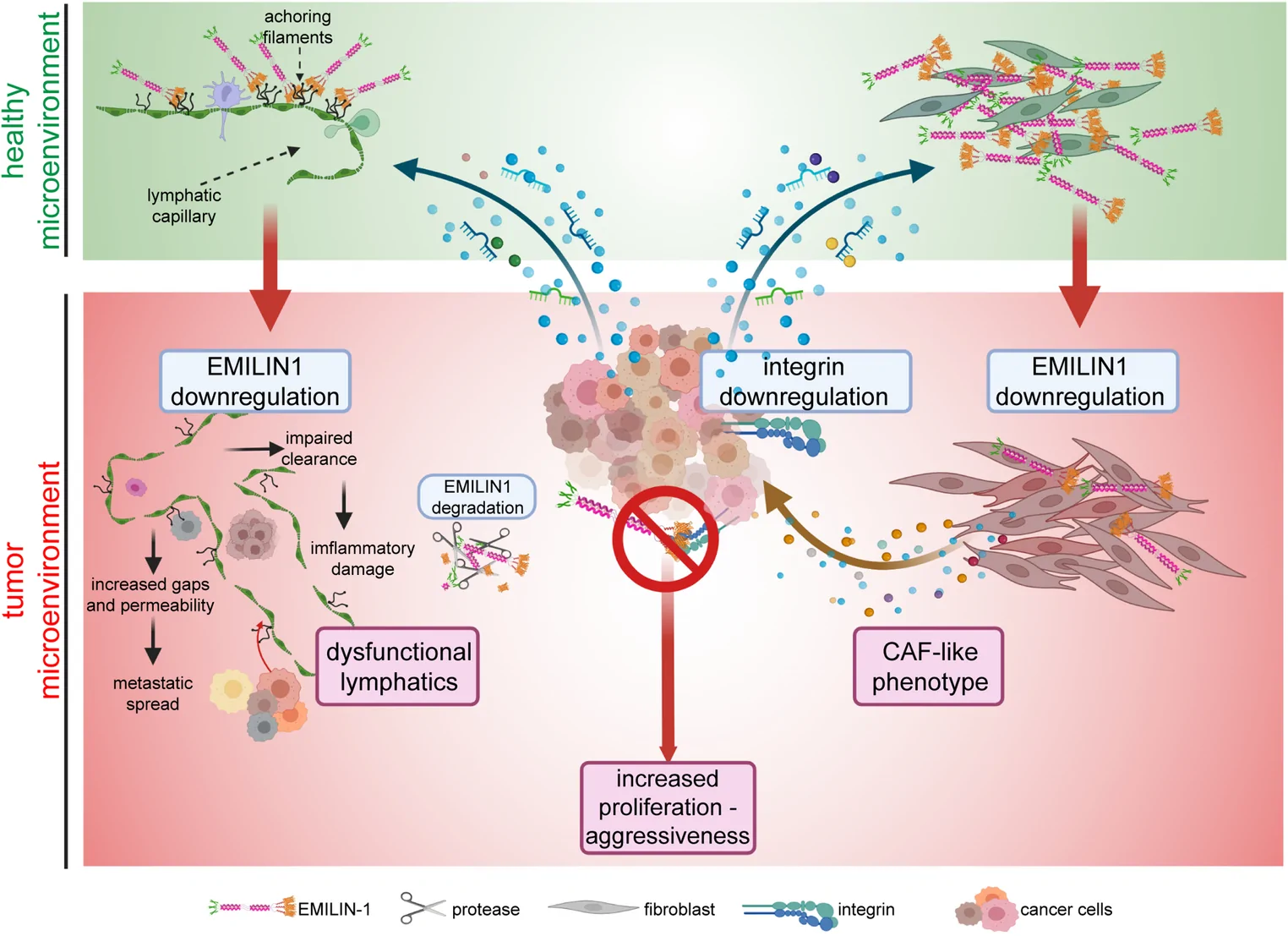
GC cells remodel the TME through dual impairment of the EMILIN-1/integrin axis. GC cells disrupt the EMILIN-1/integrin signaling axis through coordinated downregulation of stromal EMILIN-1 and epithelial α4β1/α9β1 integrins. Loss and proteolytic degradation of EMILIN-1 impair LEC function, increasing vessel permeability and inflammatory damage, while simultaneously promoting fibroblast activation toward a CAF-like phenotype. These stromal alterations enhance GC cell proliferation and invasiveness, identifying EMILIN-1 as a critical regulator of gastric tumor microenvironment homeostasis

Our study uncovers a previously unexplored mechanism by which GC cells remodel the TME through dual impairment of the EMILIN-1/integrin axis (Fig. 7). EMILIN-1, an ECM glycoprotein with anti-proliferative functions, emerges as a key stromal gatekeeper restraining epithelial transformation and fibroblast activation. Its loss, via genetic mutation, proteolysis, or transcriptional silencing, creates a permissive niche for neoplastic progression.

The increased susceptibility of E955A KI mice, expressing an EMILIN-1 mutant unable to bind α4/α9β1 integrins, highlights the importance of this interaction in maintaining stromal integrity and restraining early tumorigenesis. Consistently, selective loss of EMILIN-1 in human dysplastic lesions suggests that stromal alterations are early events in GC progression rather than passive consequences of epithelial transformation.

In vitro*,* GC cells further amplified this downregulation by reducing EMILIN-1 expression in stromal fibroblasts and LECs through soluble factors present in CM. The GC cell lines used in this study were selected to represent distinct histological and biological GC subtypes, including intestinal-type (AGS), diffuse-type (Hs746T), and metastatic GC (NCI-N87). Although some quantitative variability was observed among cell lines, all GC-derived CM consistently induced EMILIN-1 downregulation, supporting the broad relevance of this phenomenon across heterogeneous GC contexts. The observed inter-cell-line variability likely reflects the intrinsic molecular heterogeneity of GC and its associated stromal remodeling programs. A limitation of the present study is the loss of systematic analyses of cohorts of intestinal-type, diffuse-type, and metastatic gastric cancer specimens to determine whether the degree of EMILIN-1 loss varies among these subgroups and whether it correlates with clinicopathological features. This represents an important direction for future investigation.

Our findings suggest that EMILIN-1 loss is not merely a passive consequence of matrix degradation, but the result of an active tumor-driven suppression mechanism. This effect appears selective and primarily transcriptional, since other ECM components, such as fibronectin, are preserved or even upregulated under the same conditions. Interestingly, EMILIN-1 suppression was observed specifically in response to GC-derived CM, whereas CM from colon or ovarian cancer cells did not produce comparable effects, suggesting the existence of tissue-specific ECM regulatory programs linked to the inflammatory and metaplastic gastric microenvironment.

The mechanisms through which GC cells suppress EMILIN-1 expression in stromal cells remain incompletely understood. One possibility is that GC-derived inflammatory mediators and proteases may cooperatively regulate EMILIN-1 availability at both transcriptional and post-translational levels, potentially involving STAT3, NF-κB, and ERK signaling pathways. In addition, previous studies from our group demonstrated that neutrophil elastase and matrix metalloproteinases proteolytically cleave EMILIN-1 [33, 34, 36], further contributing to its loss during GC progression.

GC cells disrupt the EMILIN-1/integrin axis through a dual mechanism: suppression of stromal EMILIN-1 together with downregulation of its epithelial receptor. This coordinated loss weakens tumor stroma communication and promotes a permissive microenvironment for cancer progression. Consistently, restoration of integrin expression in GC cells re-established EMILIN-1-mediated growth suppression, supporting the functional relevance and therapeutic potential of this pathway.

Beyond epithelial regulation, EMILIN-1 loss impaired LEC tube formation and promoted fibroblast activation toward a myofibroblastic/CAF-like phenotype. Recent studies suggest that fibroblast populations involved in gastric tumorigenesis may display substantial plasticity and context-dependent functional specialization rather than fitting into rigid CAF classifications. In particular, distinct stromal populations have been identified, including PDGFRA positive telocyte-like fibroblasts, inflammatory fibroblasts, ACTA2^high^ myofibroblast-like fibroblasts, and pericyte-like populations, all coexisting within normal, metaplastic, and dysplastic gastric mucosa [37]. Moreover, analysis of the single-cell transcriptomic dataset reported by Lee et al. [38] revealed lower EMILIN1 expression in tumor-associated fibroblast populations than in non-malignant stromal fibroblasts, consistent with our findings.

Altered ECM-integrin signaling following EMILIN-1 loss may disrupt mechanotransduction pathways and activate pro-inflammatory and pro-survival cascades involved in stromal reprogramming. Accordingly, EMILIN-1-deficient fibroblasts displayed activation of AKT, ERK, NF-κB, and STAT3 signaling, enhanced CTGF expression, and increased tumor-supportive functions, including promotion of GC cell migration, clonogenicity, and invasive sprouting. Moreover, these observations raise the possibility that fibroblast activation may also influence neighboring LECs, consistent with previous reports describing a crosstalk during lymphatic remodeling and tumor progression [39]. Together, these findings support a critical role for EMILIN-1 in maintaining stromal homeostasis and restraining tumor-promoting microenvironmental remodeling.

These data also provide a mechanistic framework for interpreting our in vivo observations: the trend in mice supports this reprogramming mechanism, while the lack of significant α-SMA increase and the unchanged expression levels of α9 integrin in human dysplasia suggest EMILIN-1 loss is a very early event, initiating stromal changes before full fibroblast reprogramming is detectable. The absence of α9 integrin downregulation further indicates that integrin remodeling is likely a later event associated with malignant progression rather than with dysplastic lesions.

Taken together, these findings position EMILIN-1 as a central molecular hub in the gastric TME. Its targeted suppression by tumor cells represents a form of stromal hijacking, wherein the ECM is no longer a passive scaffold but an active regulator of tumor dynamics. This paradigm supports the idea that tumor cells evolve not only to grow autonomously but to reshape their microenvironment in ways that disarm stromal defenses.

Clinically, this opens new therapeutic avenues. Restoring EMILIN-1 function, preventing its degradation, or reactivating integrin signaling may help re-establish stromal control and restrain GC progression. Understanding how EMILIN-1 is epigenetically or post-translationally regulated may uncover actionable targets and enhance current treatments, offering a strategy that complements conventional therapies with microenvironment-focused interventions.

## Supplementary Material

Below is the link to the electronic supplementary material.


Supplementary Material 1


## Declarations

### Human and animals

All institutional and national guidelines for the care and use of laboratory animals were followed.

## References

[1] Yang W-J, Zhao H-P, Yu Y, Wang J-H, Guo L, Liu J-Y, et al. Updates on global epidemiology, risk and prognostic factors of gastric cancer. World J Gastroenterol. 2023;29:2452–68. 10.3748/wjg.v29.i16.2452. 37179585 PMC10167900

[2] Sung H, Ferlay J, Siegel RL, Laversanne M, Soerjomataram I, Jemal A, et al. Global cancer statistics 2020: GLOBOCAN estimates of incidence and mortality worldwide for 36 cancers in 185 countries. CA Cancer J Clin. 2021;71:209–49. 10.3322/caac.21660. 33538338

[3] Duan Y, Xu Y, Dou Y, Xu D. *Helicobacter pylori* and gastric cancer: mechanisms and new perspectives. J Hematol Oncol. 2025;18:10. 10.1186/s13045-024-01654-2. 39849657 PMC11756206

[4] Elhanani O, Ben-Uri R, Keren L. Spatial profiling technologies illuminate the tumor microenvironment. Cancer Cell. 2023;41:404–20. 10.1016/j.ccell.2023.01.010. 36800999

[5] Bressan GM, Daga-Gordini D, Colombatti A, Castellani I, Marigo V, Volpin D. Emilin, a component of elastic fibers preferentially located at the elastin-microfibrils interface. J Cell Biol. 1993;121:201–12. 8458869 10.1083/jcb.121.1.201 PMC2119774

[6] Danussi C, Spessotto P, Petrucco A, Wassermann B, Sabatelli P, Montesi M, et al. Emilin1 deficiency causes structural and functional defects of lymphatic vasculature. Mol Cell Biol. 2008;28:4026–39. 10.1128/MCB.02062-07. 18411305 PMC2423131

[7] Danussi C, Petrucco A, Wassermann B, Pivetta E, Modica TM, Del Bel Belluz L, et al. EMILIN1-Α4/Α9 integrin interaction inhibits dermal fibroblast and keratinocyte proliferation. J Cell Biol. 2011;195:131–45. 10.1083/jcb.201008013. 21949412 PMC3187715

[8] Danussi C, Petrucco A, Wassermann B, Modica TME, Pivetta E, Del Bel Belluz L, et al. An EMILIN1-negative microenvironment promotes tumor cell proliferation and lymph node invasion. Cancer Prev Res (Phila). 2012;5:1131–43. 10.1158/1940-6207.CAPR-12-0076-T. 22827975

[9] Capuano A, Pivetta E, Baldissera F, Bosisio G, Wassermann B, Bucciotti F, et al. Integrin binding site within the gC1q domain orchestrates EMILIN-1-induced lymphangiogenesis. Matrix Biol. 2019;81:34–49. 10.1016/j.matbio.2018.10.006. 30408617

[10] Muzzin S, Timis E, Doliana R, Mongiat M, Spessotto P. “Unraveling EMILIN-1: a multifunctional ECM protein with tumor-suppressive roles” mechanistic insights into cancer protection through signaling modulation and lymphangiogenesis control. Cells. 2025;14:946. 10.3390/cells14130946. 40643467 PMC12249408

[11] Munjal C, Opoka AM, Osinska H, James JF, Bressan GM, Hinton RB. TGF-beta mediates early angiogenesis and latent fibrosis in an Emilin1-deficient mouse model of aortic valve disease. Dis Model Mech. 2014;7:987–96. 10.1242/dmm.015255. 25056700 PMC4107327

[12] Capuano A, Vescovo M, Canesi S, Pivetta E, Doliana R, Nadin MG, et al. The extracellular matrix protein EMILIN-1 impacts on the microenvironment by hampering gastric cancer development and progression. Gastric Cancer. 2024;27:1016–30. 10.1007/s10120-024-01528-z. 38941035 PMC11335817

[13] Park J, Song S-H, Kim TY, Choi M-C, Jong H-S, Kim T-Y, et al. Aberrant methylation of integrin Alpha4 gene in human gastric cancer cells. Oncogene. 2004;23:3474–80. 10.1038/sj.onc.1207470. 14990990

[14] Kang GH, Lee S, Cho N-Y, Gandamihardja T, Long TI, Weisenberger DJ, et al. DNA methylation profiles of gastric carcinoma characterized by quantitative DNA methylation analysis. Lab Invest. 2008;88:161–70. 10.1038/labinvest.3700707. 18158559

[15] Kim JH, Jung EJ, Lee HS, Kim MA, Kim WH. Comparative analysis of DNA Methylation between primary and metastatic gastric carcinoma. Oncol Rep. 2009;21:1251–9. 10.3892/or_00000348. 19360301

[16] Spessotto P, Cervi M, Mucignat MT, Mungiguerra G, Sartoretto I, Doliana R, et al. Β1 integrin-dependent cell adhesion to EMILIN-1 is mediated by the gC1q domain*. J Biol Chem. 2003;278:6160–7. 10.1074/jbc.M208322200. 12456677

[17] Capuano A, Pivetta E, Sartori G, Bosisio G, Favero A, Cover E, et al. Abrogation of EMILIN1-Β1 integrin interaction promotes experimental colitis and colon carcinogenesis. Matrix Biol. 2019;83:97–115. 10.1016/j.matbio.2019.08.006. 31479698

[18] Pivetta E, Capuano A, Vescovo M, Scanziani E, Cappelleri A, Rampioni Vinciguerra GL, et al. EMILIN-1 deficiency promotes chronic inflammatory disease through TGFβ signaling alteration and impairment of the gC1q/Α4β1 integrin interaction. Matrix Biol. 2022;111:133–52. 10.1016/j.matbio.2022.06.005. 35764213

[19] Favero A, Segatto I, Capuano A, Mattevi MC, Rampioni Vinciguerra GL, Musco L, et al. Loss of the extracellular matrix glycoprotein EMILIN1 accelerates Δ16HER2-driven breast cancer initiation in mice. NPJ Breast Cancer. 2024;10:5. 10.1038/s41523-023-00608-0. 38184660 PMC10771445

[20] Saenz, J.B.; Burclaff, J.; Mills, J.C. Modeling Murine Gastric Metaplasia Through Tamoxifen-Induced Acute Parietal Cell Loss. In *Gastrointestinal Physiology and Diseases: Methods and Protocols*; Ivanov, A.I., Ed.; Springer: New York, NY, 2016;. 329–339 ISBN 978–1–4939–3603–8. 10.1007/978-1-4939-3603-8_28 PMC5221414 27246044

[21] Petersen CP, Mills JC, Goldenring JR. Murine models of gastric corpus preneoplasia. Cell Mol Gastroenterol Hepatol. 2017;3:11–26. 10.1016/j.jcmgh.2016.11.001. 28174755 PMC5247421

[22] Tissino E, Pivetta E, Capuano A, Capasso G, Bomben R, Caldana C, et al. Elastin MIcrofibriL INterfacer1 (EMILIN-1) is an alternative prosurvival VLA-4 ligand in chronic lymphocytic leukemia. Hematol Oncol. 2022;40:181–90. 10.1002/hon.2947. 34783040

[23] Modica TME, Maiorani O, Sartori G, Pivetta E, Doliana R, Capuano A, et al. The extracellular matrix protein EMILIN1 silences the RAS-ERK pathway via Α4β1 integrin and decreases tumor cell growth. Oncotarget. 2017;8:27034–46. 10.18632/oncotarget.15067. 28177903 PMC5432316

[24] Kabadi AM, Ousterout DG, Hilton IB, Gersbach CA. Multiplex CRISPR/Cas9-based genome engineering from a single lentiviral vector. Nucleic Acids Res. 2014;42:e147. 10.1093/nar/gku749. 25122746 PMC4231726

[25] Spessotto, P.; Giacomello, E.; Perris, R. Fluorescence Assays to Study Cell Adhesion and Migration In Vitro. In *Extracellular Matrix Protocols*; Streuli, C.H., Grant, M.E., Eds.; Methods in Molecular Biology^TM^; Humana Press: Totowa, NJ, 2000. 321–343 ISBN 978–1–59259–063–6. 10.1385/1-59259-063-2:321 10840800

[26] Danussi C, Del Bel Belluz L, Pivetta E, Modica TM, Muro A, Wassermann B, et al. EMILIN1/Alpha9beta1 integrin interaction is crucial in lymphatic valve formation and maintenance. Mol Cell Biol. 2013;33:4381–94. 10.1128/MCB.00872-13. 24019067 PMC3838180

[27] Capuano A, Fogolari F, Bucciotti F, Spessotto P, Nicolosi PA, Mucignat MT, et al. The Α4β1/EMILIN1 interaction discloses a novel and unique integrin-ligand type of engagement. Matrix Biol. 2018;66:50–66. 10.1016/j.matbio.2017.10.001. 29037761

[28] Rojas A, Araya P, Gonzalez I, Morales E. Gastric tumor microenvironment. Adv Exp Med Biol. 2020;1226:23–35. 10.1007/978-3-030-36214-0_2. 32030673

[29] Wei J, Wang M, Li G. Cancer-associated fibroblasts, and clinicopathological characteristics and prognosis of gastric cancer: a systematic review and meta-analysis. Front Oncol. 2023. 10.3389/fonc.2023.1048922. 36874089 PMC9981791

[30] Choi K-M, Kim B, Lee S-M, Han J, Bae H-S, Han S-B, et al. Characterization of gastric cancer-stimulated signaling pathways and function of CTGF in cancer-associated fibroblasts. Cell Commun Signal. 2024;22:8. 10.1186/s12964-023-01396-7. 38167009 PMC10763493

[31] Ozmen E, Demir TD, Ozcan G. Cancer-associated fibroblasts: protagonists of the tumor microenvironment in gastric cancer. Front Mol Biosci. 2024;11:1340124. 10.3389/fmolb.2024.1340124. 38562556 PMC10982390

[32] Goldenring JR, Ray GS, Coffey RJ, Meunier PC, Haley PJ, Barnes TB, et al. Reversible drug-induced oxyntic atrophy in rats. Gastroenterology. 2000;118:1080–93. 10.1016/s0016-5085(00)70361-1. 10833483

[33] Pivetta E, Wassermann B, Del Bel Belluz L, Danussi C, Modica TM, Maiorani O, et al. Local inhibition of elastase reduces EMILIN1 cleavage reactivating lymphatic vessel function in a mouse lymphoedema model. Clin Sci (Lond). 2016;130:1221–36. 10.1042/CS20160064. 26920215 PMC4888021

[34] Maiorani O, Pivetta E, Capuano A, Modica TM, Wassermann B, Bucciotti F, et al. Neutrophil elastase cleavage of the gC1q domain impairs the EMILIN1-Alpha4beta1 integrin interaction, cell adhesion and anti-proliferative activity. Sci Rep. 2017;7:39974. 10.1038/srep39974. 28074935 PMC5225433

[35] Corriden R, Hollands A, Olson J, Derieux J, Lopez J, Chang JT, et al. Tamoxifen augments the innate immune function of neutrophils through modulation of intracellular ceramide. Nat Commun. 2015;6:8369. 10.1038/ncomms9369. 26458291 PMC4610010

[36] Pivetta E, Danussi C, Wassermann B, Modica TM, Del Bel Belluz L, Canzonieri V, et al. Neutrophil elastase-dependent cleavage compromises the tumor suppressor role of EMILIN1. Matrix Biol. 2014;34:22–32. 10.1016/j.matbio.2014.01.018. 24513040

[37] Contreras-Panta EW, Choi E, Goldenring JR. The fibroblast landscape in stomach carcinogenesis. Cell Mol Gastroenterol Hepatol. 2024;17:671–8. 10.1016/j.jcmgh.2024.02.001. 38342299 PMC10957461

[38] Lee HJ, Kwak Y, Na YS, Kim H, Park MR, Jo JY, et al. Proteomic heterogeneity of the extracellular matrix identifies histologic subtype-specific fibroblast in gastric cancer. Mol Cell Proteomics. 2024;23:100843. 10.1016/j.mcpro.2024.100843. 39305996 PMC11526087

[39] Wei W-F, Zhou H-L, Chen P-Y, Huang X-L, Huang L, Liang L-J, et al. Cancer-associated fibroblast-derived PAI-1 promotes lymphatic metastasis via the induction of EndoMT in lymphatic endothelial cells. J Exp Clin Cancer Res. 2023;42:160. 10.1186/s13046-023-02714-0. 37415190 PMC10324144

