# Supplementary material for: Loss of EMILIN-1/integrin axis drives microenvironmental reprogramming in gastric tumorigenesis
Source: Gastric Cancer. 2026 Jul 20;29(5):987–1000. doi: 10.1007/s10120-026-01781-4 (PMC13524978; doi:10.1007/s10120-026-01781-4)
Supplement: Supplementary file 1 [file 10120_2026_1781_MOESM1_ESM.docx]

**SUPPLEMENTARY MATERIAL**

**Supplementary Tables**

**Table 1. List of the antibodies used in this study**

| ANTIGEN | Type and application | COMPANY | ID |
| --- | --- | --- | --- |
| α-SMA | Mouse monoclonal, Cy3™ - IF | Sigma-Aldrich | C6198 |
| α-SMA | Rabbit monoclonal to α-SMA - WB | Abcam | ab124964 |
| α-SMA | Rabbit monoclonal antibody to α-SMA- IHC-P, ICC/IF | Abcam | ab150301 |
| α-SMA | Mouse monoclonal, clone 1A4, IHC | Sigma-Aldrich Cell Marque™ | 202M-9 |
| EMILIN-1 | Rabbit polyclonal antibody to human EMILIN-1- WB and IF | Homemade or Abcam | [1] or ab243324 |
| FIBRONECTIN | Rabbit polyclonal - WB | GeneTex | GTX112794 |
| INTEGRIN α4 | Rabbit monoclonal antibody - WB | Cell Signaling | 8440 |
| INTEGRIN α4 | Mouse monoclonal antibody - Clone P1H4 - blocking antibody | Millipore (Sigma) | MAB16983Z |
| INTEGRIN α4 | Rabbit polyclonal antibody - IHC-P | Abcam | ab202969 |
| INTEGRIN α9 | Rabbit recombinant monoclonal antibody antibody - IHC-P | Abcam | ab140599 |
| Phospho-NF-Kb p65(Ser 536) | Rabbit monoclonal antibody - Clone 93H1 - WB | Cell Signaling | 3033 |
| NF-Kb p65 | Rabbit monoclonal antibody - Clone C-20 - WB | Santa Cruz | sc372 |
| Phospho-PDGFRβ (tyr751) | Mouse monoclonal antibody - Clone 88H8 - WB | Cell Signaling | 3166 |
| PDGFRβ | Mouse monoclonal antibody - Clone 42G12 - WB | Abcam | ab69506 |
| Phospho-p44/42 MAPK (Erk1/2) (Thr202/Tyr204) | Rabbit polyclonal antibody - WB | Cell Signaling | 9101 |
| p44/42 MAPK (Erk1/2) | Rabbit polyclonal antibody - WB | Cell Signaling | 9102 |
| VINCULIN | Goat polyclonal - WB | Santa Cruz | Sc-7649 |
| GAPDH | Mouse monoclonal antibody -WB | Millipore | MAB374 |

**Table 2. List of patients with dysplasia analyzed in this study**

|  | sex | age | sample type | clinical context |
| --- | --- | --- | --- | --- |
| patient 1 | M | 57 | small biopsy | Fundic gland polyp in the context of FAP |
| patient 2 | M | 81 | mucosectomy | sporadic dysplasia |
| patient 3 | M | 58 | small biopsy | hyperplastic polip in the context of Lynch syndrome |
| patient 4 | F | 73 | mucosectomy | atrophic gastritis |
| patient 5 | F | 62 | small biopsy | atrophic gastritis |
| patient 6 | F | 73 | mucosectomy | atrophic gastritis |
| patient 7 | F | 83 | mucosectomy | atrophic gastritis |
| patient 8 | M | 52 | small biopsy | FAP |
| patient 9 | M | 48 | small biopsy | FAP associated to hyperplasia |
| patient 10 | F | 78 | small biopsy | Fundic gland polyp in the context of FAP |

**Table 3. List of primers used for RT-PCR analyses**

| GENE | FORWARD PRIMER (5’ > 3’) | REVERSE PRIMER (5’ > 3’) |
| --- | --- | --- |
| *ACTA2* | TATCCCCGGGACTAAGACGG | CACCATCACCCCCTGATGTC |
| *EMILIN1* | TCCCCAAAGCATCATGTACCG | CACAGTCATCGCCCCCATAA |
| *FIBRONECTIN* | GTGTGATCCCGTCGACCAAT | CGACAGGACCACTTGAGCTT |
| *GAPDH* | GAGAGACCCTCACTGCTG | GATGGTACATGACAAGGTGC |
| *CTGF* | CTCGTGCTGGACGGCTG | AGCACCATCTTTGGCGGTG |
| *COL1A1* | GGAATGAAGGGACACAGAGGTT | AGTAGCACCATCATTTCCACGA |
| *FAP* | CATCTGGAAAAATGAAGACTTGGGT | AGGGCGTAAGACAATGCACA |
| *PDGFRβ* | GGAGAGGGCAGTAAGGAGGA | AAGCCGCATGGTGTCCTTG |

**Table 4. Sequences used to generate KO fibroblasts**

| Gene |  | Sequence 5’-3’ |
| --- | --- | --- |
| EMILIN1 | RS_CRISPR_132_S | ttgtttgTGGGCGGGATGAGTCTCTGA |
| EMILIN1 | RS_CRISPR_132_AS | aaacTCAGAGACTCATCCCGCCCAcaa |
| EMILIN1 | RS_CRISPR_133_S | caccgCTGTGGAGCGCCCCGCCATG |
| EMILIN1 | RS_CRISPR_133_AS | aaacCATGGCGGGGCGCTCCACAGc |
| EMILIN1 | RS_CRISPR_134_S | tcccaAGAGTCTGGATCCCAGCCCG |
| EMILIN1 | RS_CRISPR_134_AS | aaacCGGGCTGGGATCCAGACTCTt |
| EMILIN1 | RS_CRISPR_135_AS | cctcgCTGCTGTGTCCGCTAATGCA |
| EMILIN1 | RS_CRISPR_135_S | aaacTGCATTAGCGGACACAGCAGc |

**Table 5. Sequences used to screen Bj-5ta fibroblasts**

| PRIMER | Sequence 5’-3’ |
| --- | --- |
| MiSeq_ F | tcgtcggcagcgtcagatgtgtataagagacagGGGGGCTATCAGAAGGAAAC |
| MiSeq_ R | gtctcgtgggctcggagatgtgtataagagacagCCTAGGTGACCCAAGGAACA |
| EMILIN1_KOscreen_ F | ACCCTCTGGAGCTGCTACCT |
| EMILIN1_KOscreen_ R | CCCCACTGGAACCTGTGTA |

**Supplementary methods**

**3D tumor spheroid model.**

GC cells and fibroblasts were stained separately with DiO and DiI, respectively, for 15 min at 37°C. The cells were then mixed at a 2:1 ratio and suspended in 0.24% methylcellulose diluted in F-12K medium. Twenty-microliter hanging drops were placed on inverted lids of culture dishes containing sterile water to maintain humidity and incubated at 37°C in a 5% CO₂ atmosphere for 48 h to promote cell aggregation. Following incubation, the hanging drops were collected and centrifuged at 20 × g for 3 min. The resulting cell pellets were resuspended in Matrigel (3 mg/mL) and seeded into 24-well plates for further incubation. After an additional 48 h, samples were imaged using a Thunder imaging system (Leica Microsystems), and image acquisition was performed using Thunder Viewer software.

**Supplementary Figures**

**Fig. S1. Integrin expression profiles in GES-1 and AGS cells.** Flow cytometry (FACS) analysis of integrin surface expression in the non-transformed gastric epithelial cell line GES-1 (red histograms) and the GC cell line AGS (green histograms).

**Fig. S2. Lack of integrin α4β1 expression in GC cells.** FACS analysis of integrin surface expression in three GC cell lines: Hs746T (A), NCI-N87 (B), and MKN45 (C). The red box highlights the absence of both α4 and α9 integrin subunits. (D) Proliferation measured with xCELLigence technology of MKN45 cells in response to the presence of recombinant gC1q protein.

**Fig. S3. EMILIN-1 is selectively downregulated in the gastric tumor microenvironment.** (A) RT-PCR analysis showing EMILIN1 expression in primary fibroblasts and lymphatic endothelial cells (LECs), but not in GC cell lines. (B–C) Immunofluorescence staining (B) and mRNA expression analysis (C) of fibronectin (used as a control) in dermal fibroblasts cultured with conditioned media (CM) from GC cell lines. Quantification of the fibronectin signal (green) is provided. (D) EMILIN1 mRNA levels in dermal fibroblasts cultured in the presence of colon (left) or ovarian (right) cancer cells. Results are expressed as the mean ± SD of independent experiments (dots in the graphs). ***P*<0.01. Scale bar: 50 μm.

**Fig. S4. Validation and characterization of EMILIN-1 knockout fibroblasts.** Stable EMILIN-1 knockout (KO) fibroblasts were generated using CRISPR/Cas9 with guide RNAs targeting the EMILIN1 locus to induce frameshift deletions. KO validation was performed via: (A) immunofluorescence and (B) Western blot analysis confirming the absence of EMILIN-1 protein. (C) qRT-PCR analysis shows a significant increase of mRNA expression of *ACTA2* (encoding α-SMA) and *CTGF* in KO Bj-5ta clones. *GAPDH* was used as a reference gene. **P*<0.05. Scale bar: 50 μm.

Fig. S5. **Figure S5. EMILIN-1-deficient fibroblasts promote AGS cell migration.** (A) Haptotactic migration of AGS GC cells toward different ECM components, including collagen I (Col I), collagen IV (Col IV), fibronectin (FN), and laminin-1 (Lam-1). Representative images show DiI-labeled AGS cells (red) after 20 h of migration. (B) Quantification of AGS cell haptotaxis toward fibronectin in the presence of conditioned medium (CM) derived from wild-type (WT) or EMILIN-1 knockout (KO) BJ-5ta fibroblast clones (n = 4). Data represent the mean number of migrated cells quantified from more than three independent 20× microscopic fields per replicate. (C, D) CM from EMILIN-1 KO fibroblasts enhances AGS cell migration in a wound-healing assay performed using Ibidi inserts. (C) Representative images of wound closure at 0 and 20 h. (D) Quantification of the percentage of wound closure after 20 h. Fine modulo***P*<0.01, ****P*<0.001, *****P*<0.0001.

Fig. S5. **Figure S6. EMILIN-1-deficient fibroblasts promote Hs746T cell migration and invasive sprouting.** (A) Haptotactic migration of Hs746T GC cells toward different ECM components, including collagen I (Col I), collagen IV (Col IV), fibronectin (FN), and laminin-1 (Lam-1). Representative images show DiI-labeled Hs746T cells (red) after 20 h of migration. (B) Quantification of Hs746T cell haptotaxis toward collagen IV in the presence of conditioned medium (CM) derived from wild-type (WT) or EMILIN-1 knockout (KO) BJ-5ta fibroblasts. Data represent the mean number of migrated cells quantified from more than three independent 20× microscopic fields per replicate. (C) CM from EMILIN-1 KO fibroblasts enhances Hs746T cell migration in a wound-healing assay. Two independent experiments are reported, and wound closure over time is quantified in the graph. (D) 3D spheroids composed of Hs746T cells (DiO-labelled, green) co-cultured with WT or KO BJ-5ta fibroblasts (DiI-labelled, red) were embedded in Matrigel and cultured for 48 h. Representative images acquired with the Thunder Viewer System (Leica Microsystems) are shown. (E) Graphs report quantification of spheroid size and sprouting-associated invasive area. Fine modulo

**P*<0.05, ***P*<0.01, NS, not significant.

**Fig. S7. Histological changes following intraperitoneal tamoxifen treatment. (A)** Representative H&E-stained gastric sections displaying normal oxyntic mucosa (left panel, score 0) and severe depletion of parietal cells (right panel, score 3); the same H&E-stained images observed under a fluorescence microscope are also shown. (B) Quantification of morphological alterations in the gastric mucosa (fundic mucosa, F and fundic mucosa at the border with the forestomach, *margo plicatus,* MP) of wild-type (WT) and EMILIN-1 mutant (KI) mice after intraperitoneal tamoxifen administration. KI mice (n = 15) displayed increased nuclear debris within the glandular lumen and pronounced regenerative hyperplasia compared to WT controls (n = 6).

**Fig. S8. Loss of EMILIN-1 in gastric dysplasia.** (A) Semi-quantitative H-score analysis (range 0-300) of EMILIN-1 expression in paired normal and dysplastic regions (n=10 patients). (B) Quantification score (range 0-5) for α-SMA expression in paired normal and dysplastic regions (n=10 patients). (C) Quantification score (range 0-5) for α9 integrin expression in paired normal and dysplastic regions (n=10 patients). Data are presented as individual paired points. ***P*<0.01 (paired t-test); NS, not significant.

**Fig. S9. Alterations of the EMILIN1-integrin axis in gastric cancer and association with clinical outcome**.

(A) The graphs represent the gene expression analysis of *ITGA4* (alpha4 integrin, left) and ITGA9 (alpha9 integrin, right) evaluated with the GEO dataset GSE66229 (normal, n=100; tumor, n=300) *****P*<0.0001, NS, not significant. (B) Graph showing *ITGA9* expression in different tumors and the corresponding normal tissue as assessed with the TNMplot tool (<https://tnmplot.com/analysis>) [2]. The tumors with statistically different values are indicated in the graph with an asterisk and in red. For the stomach the number of samples analyzed (N = normal; T = tumor) is also indicated. (C) Targetgram analysis showing the expression of the investigated genes (EMILIN1, ITGA9 and TGFB1) in GC. The size of the segments is proportional to the mean values of gene expression, whereas length of the dashed lines corresponds to the median values in each type. (D) Kaplan-Meier plot estimating the overall survival (OS) (left), the first progression (FP) (middle) and post-progression survival (PPS) (right) of GC patients stratified for EMILIN1 expression using the KM Plotter dataset (https://kmplot.com). HR = Hazard Ratio. The Confidence interval is reported between brackets. The *P* value was calculated using the log-rank test. (E) Median survival (months) for patients with high and low EMILIN1 expression in gastric cancer. Overall survival (OS) and first progression (FP) are reported for all cases and stratified by stage. *P* values were calculated using the log-rank test.

**References**

1. Spessotto P, Cervi M, Mucignat MT, Mungiguerra G, Sartoretto I, Doliana R, et al. β1 Integrin-dependent Cell Adhesion to EMILIN-1 Is Mediated by the gC1q Domain*. *Journal of Biological Chemistry*. 2003;278:6160–7. https://doi.org/10.1074/jbc.M208322200

2. Bartha Á, Győrffy B. TNMplot: An enhanced platform for pharmacological target identification through cross-stage and pan-cancer gene expression analysis. *British Journal of Pharmacology*. 2026;183:2648–59. https://doi.org/10.1111/bph.70390
